# Prevalence of Rare Genetic Variations and Their Implications in NGS-data Interpretation

**DOI:** 10.1038/s41598-017-09247-5

**Published:** 2017-08-29

**Authors:** Yangrae Cho, Chul-Ho Lee, Eun-Goo Jeong, Min-Ho Kim, Jong Hui Hong, Younhee Ko, Bomnun Lee, Gilly Yun, Byong Joon Kim, Jongcheol Jung, Jongsun Jung, Jin-Sung Lee

**Affiliations:** 1 Syntekabio Incorporated, Techno-2ro B-512, Yuseong-gu, Daejeon, 34025 Republic of Korea; 2 DFTBA, CALS, Chonnam National University, Gwangju, 61186 Republic of Korea; 3 Department of Clinical Genetics, Department of Pediatrics, Yonsei University College of Medicine, Seoul, 03722 Republic of Korea; 4 Department of Biomedical Engineering, Hankuk University of Foreign Studies, Yongin-si, Gyeonggi-do 17035 Republic of Korea

## Abstract

Next-generation sequencing (NGS) technology has improved enough to discover mutations associated with genetic diseases. Our study evaluated the feasibility of targeted NGS as a primary screening tool to detect causal variants and subsequently predict genetic diseases. We performed parallel computations on 3.7-megabase-targeted regions to detect disease-causing mutations in 103 participants consisting of 81 patients and 22 controls. Data analysis of the participants took about 6 hours using local databases and 200 nodes of a supercomputer. All variants in the selected genes led on average to 3.6 putative diseases for each patient while variants restricted to disease-causing genes identified the correct disease. Notably, only 12% of predicted causal variants were recorded as causal mutations in public databases: 88% had no or insufficient records. In this study, most genetic diseases were caused by rare mutations and public records were inadequate. Most rare variants, however, were not associated with genetic diseases. These data implied that novel, rare variants should not be ignored but interpreted in conjunction with additional clinical data. This step is needed so appropriate advice can be given to primary doctors and parents, thus fulfilling the purpose of this method as a primary screen for rare genetic diseases.

## Introduction

Timely diagnosis of genetic diseases could give newborns a chance to live healthy, independent lives as opposed to lives of disability and frequent or prolonged hospital visits. Tandem mass spectrometry, or MS/MS, is used in California to screen for selected genetic disorders of metabolism, endocrine disruptions, humoral diseases and cystic fibrosis^1^. This same method is used to screen for six metabolic diseases in the Republic of Korea (ROK) and is free to all newborns. In addition, most medical institutes, including public clinics and private hospitals, provide similar screening services for over 50 diseases detectable by MS/MS. Primary screening of genetic disorders by this method is fast and economical, but the false positive rate is over 90% according to a report by the President’s Council on Bioethics in the USA^2^. The trend is similar in the ROK, with false positive rates above 90% (T. Kim, personal communication).

Despite false positives, MS/MS is used to screen for neonatal metabolic diseases because early detection is critical for preventative treatment and preemptive management of devastating neonatal genetic diseases. Genetic screening is used as a secondary confirmatory test after a primary screening with MS/MS. Genetic testing was generally performed one gene at a time using Sanger sequencing. Recently, next-generation sequencing (NGS) was used to scan many genes at the same time^2–8^. Due to its high cost or labor intensity, this test was limited to patients with clinical indications or family history^2^.

With the advancement of NGS technology, the cost per base for determining the nucleotide sequence of many genes at the same time was dramatically reduced. The power of NGS technology made whole-genome sequencing (WGS) or whole-exome sequencing (WES) possible and showed promise for early screening of genetic disorders in newborns and for pediatric diagnostic medical care^9–13^. If NGS is to be used as a primary means for screening genetic disorders in newborns, however, several technical issues need to be addressed. They include noninvasiveness, reasonable turnaround time, affordable cost, and reasonable interpretation of variants. Dry Blood Spot (DBS) has been used routinely to extract a sufficient amount of DNA for subsequent NGS use. Collecting DBS is minimally invasive to newborns, extracts less than 75 µl of blood, and the sample is easy to store for future use^2, 6, 14, 15^. Turnaround time from DBS collection to NGS analysis can be as short as 5 days (equivalent to 105 hours) This is an acceptable interval for a rapid neonatal screening test^2^. The associated cost, however, would be too high for routine primary screening. The main expense is the prohibitive fixed cost of high-throughput NGS sequencing and subsequent data analysis, although technological innovation has reduced the cost per base.

Our goals in this study were to test a high-performance computing platform to analyze targeted NGS (TNGS) *in silico*; to assess the ability to discover variants with little public information but associated with specific genetic disorders; and to discuss issues regarding the interpretation of rare variants, with insufficient public records, in targeted genes associated with genetic disorders.

## Results

### Speed of Data Analysis

**Figure 1:**
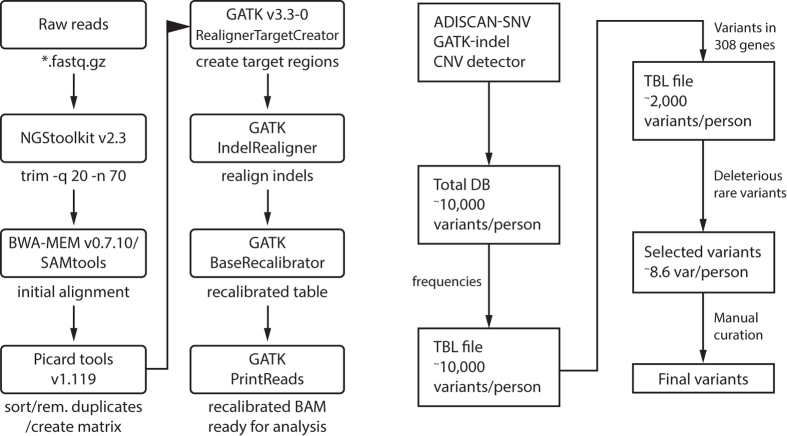
Work flow for generation of a binary alignment matrix (BAM). These files were used in subsequent genotyping and variant discovery with four separate variant calling methods.

Target regions for intended sequencing were 3.7 Mb and included all positions in 5,770 exons of 362 genes, plus 13,000 positions with biological importance. The entire process was performed as part of a validation to benchmark the results, optimize selection logic, and estimate computation time from the BAM file to the final results. When one node of a computer was used, it took about one hour to produce a recalibrated BAM file and another four hours to create a file of candidate genes associated with rare genetic diseases. This file included annotated information on the frequency at which variants occurred in four different populations and the functional importance of sequence variations. It would take 58 days to analyze 103 samples if one node was used, but it took only 5 hours to complete the same analysis using 200 nodes of a server-class computer.(Fig. 1, Table 1).

**Table 1:** Summary of specifications for the computation time for 103 DNA samples versus one DNA sample.

Number of samples	Sample Size	Computation time		
		FASTQ to BAM	BAM to VCF	Total (Hour)
1	1.0GB	0:45	2:30	3:15
1	1.5GB	1:05	3:30	4:35
1	2.8GB	2:03	6:50	8:53
103	165GB (1.5 GB ea.)	1:05	3:30	5:00*

OS: CentOS6.5, CPU: Intel Xeon 2Socket E5520 2.3 GHz 4Core × 200Node (total = 1,600 Cores), Disk Drive: MAHA distributed parallel file system for diagnosis (MAHA-FsDx: 1.4 PetaByte). *The actual time required for the analysis of 103 samples on 200 nodes of a MAHA-FsDx, parallel computer was 5 hours, instead of 4 hours 35 minutes.

### NGS Sequencing Statistics

**Figure 2:**
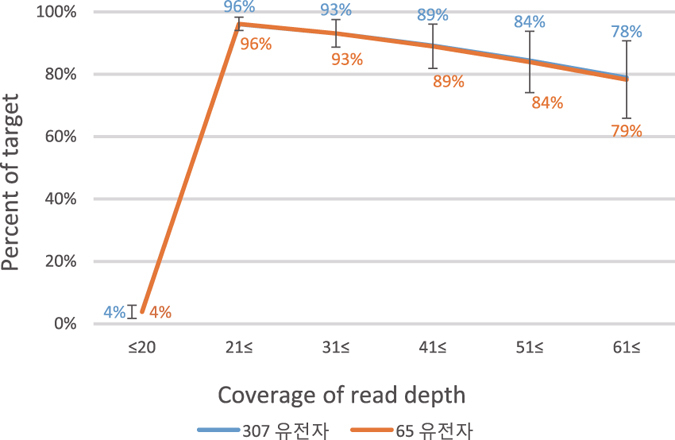
Read depth distribution for the target calculated by the formula: sum of read numbers in each bracket divided by the number of total positions. Read depth distribution for the target was similar for both the 65 and 307 target genes. The blue line almost overlapped the orange line.

We scanned the 3.7-Mb regions and discovered that all variants in the regions were sequenced at an average depth of 161× reads, but focused our analysis on 307 of the 362 genes in 1.38 Mb. We excluded 55 of the 362 genes because they were not associated with rare genetic diseases, but with other common features such as blood and HLA types (Supplementary Table S1). Sequence depth averaged 128× for the 307 genes in subsequent data interpretations (Table 2, Supplementary Table S2). Capture performance data indicated that 96% of the targeted bases were covered at a minimum depth of 20× reads (Fig. 2). The remaining 4% of the regions were either not sequenced or were inadequately aligned. We did not pursue the reasons for the missing sequences. NGS sequencing can be affected by various factors such as poor capturing due to high G/C content or repetitive sequences. Read depth for 79% of the targeted regions exceeded a minimum coverage depth of 61× (Fig. 2). Sequence variants initially were determined for all positions regardless of read depths. An average of 11,463 (s.d. 199) variants were detected among the 3.7-Mb positions included in the panel, and 1,186 (s.d. 57.2) of the 11, 463 variants occurred within the 307 genes. Because several authors indicated a minimum read depth coverage of 20× for accurate genotyping of a base^16, 17^, we used 20× in our downstream analysis to discover sequence variants. Because sequence coverage exceeded 30× for 93% of all samples we also used 30× with an expectation that it would minimize calling errors. There were no differences, however, between the 20× and 30× sets in the final sets of variants.

**Table 2:** Statistics for sequence-read coverages and rare variants leading to the causal variants for rare genetic diseases in each patient.

	Captured regions	307 genes	211 genes	65 genes
Sum of reads on target regions	575 Mb	138 Mb	109 Mb	43 Mb
On target rates^1^	30%	7.20%	5.20%	2.20%
Length of target regions	3.7 Mb	1.3 Mb	0.9 Mb	0.35 Mb
Mean read depth^2^	161×	128×	120×	127×
Number of variants	NA	1186	762	271
Number of rare variants with probable deleterious effects	NA	8.6	6.2	3.4
Number of disease candidates	NA	3.6	2.7	1.8

^1^[(Sum of reads on target regions)/(sum of all reads, 2.1 Gigabytes)] × 100. ^2^(Sum of reads on target regions)/(length of target regions).

### Patient Selection

The study group was comprised of 81 patients with disease-causing mutations, 10 patients with carrier mutations, and 12 negative controls. For the 81 patients, diseases were diagnosed based on symptoms and other test results. Causal mutations were confirmed by Sanger sequencing prior to or during this study. Information on the causal mutations was not provided to the analysis team initially. This allowed the team to objectively assess the ability of NGS to discover causal variants.

### Optimization of Discovery Process/Orientation to Data

Results for base calls from the NGS workflow were compared with the results of Sanger sequencing in other studies^2, 3, 8, 18, 19^. We randomly selected 25 of the 103 patients during the initial discovery stage of variants *in silico* (Table 3, Supplementary Table S3) to optimize our informatics approach for discovering clinically important variants. There were 23 patients with predefined causative mutations for genetic disorders and two patients with variants but no causative mutations. This information was not disclosed to the analysis team, who suspected that all subject patients had causative mutations. Filtering the causal genetic variants from >99% of benign variants was based on information about the functional importance predicted by PolyPhen (ver. 2.2.2) and their frequency among healthy populations for each variant. Each individual had ~2,000 variants within the 307 genes and PolyPhen predicted an average of 75 variants with damaging effects. Screening by minor allele frequencies below 4% among general populations left on average 8.6 candidates as causal variants. Through manual curation, we identified 29 variants in 23 samples and missed 3 variants in 2 samples. The three variants were discovered initially by targeted NGS (TNGS), but inadvertently excluded during a further selection procedure because their frequency was above 5% among healthy people. These were polymorphic nucleotide sequences and served as negative controls. In conclusion, all predefined variants were rediscovered using TNGS, but three variants were excluded during the final selection stage because they were unlikely causal mutations of genetic diseases based on their high frequencies among healthy populations.

**Table 3:** Call for disorders based on nucleotide sequence variations detected by next-generation sequencing

Patient #	Gene	Variants	Variant type	Variant call	Heredity^1^
**Known single nucleotide variant (SNV)**					
37	FGFR3	(Gly382Arg (c.1138G > A, het)	rs28931614	Yes	AD
**71**	**PAH**	**p.Arg243Gln (c.728G > A)**	**rs62508588**	**Yes**	**AR**
**71**	**PAH**	**c.442-1G > A**	**rs62514907**	**Yes**	**AR**
83	RB1	c.1215 + 1G > A	rs587776783	Yes	AD
89	SOS1	p.Arg552Gly(c.1654A > G)	rs137852814	Yes	AD
102	WT1	p.Arg462Trp(c.1384 C > T, het)	rs121907900	Yes	AD
**Introduction of stop codon (nonsense mutation)**					
**1**	**ABCA12**	p.Arg2482*(c.7444 C > T)	**stop**	**Yes**	**AR**
**1**	**ABCA12**	p.Arg1950*(c.5848 C > T)	**stop**	**Yes**	**AR**
46	GCH1	p.Glu242*(c.724 G > , het)	stop	Yes	AD
56	JAG1	p.Tyr434* (c.1302 C > A, het)	stop	Yes	AD
62	NF1	p.Gln554*(c.1660C > T)	stop	Yes	AD
84	RB1	p.Arg272* (c.814 A > T, het)	stop	Yes	AD
**Insertion and deletion (indel)**					
34	FBN1	c.1698_1712del15(exon13, het)	ins	Yes	AD
55	JAG1	c.1 720 + 1dupG(intron13, het)	del	Yes	AD
**100**	**VPS33B**	**c.740_741delAT(exon10,het)**	**del**	**Yes**	**AR**
**100**	**VPS33B**	**c.403 + 2 T > A(intron6,het)**	**ins**	**Yes**	**AR**
**Single nucleotide variant (SNV) without public records**					
**10**	**ARSA**	**p.Cys493Ser (c.1478G > C,het)**	**SNV**	**Yes**	**AR**
**10**	**ARSA**	**p.Asp411Gly (c.1232A > G,het)**	**SNV**	**Yes**	**AR**
24	COL1A2	c.595-1G > C	SNV	Yes	AD
26	COMP	p.Asp376His (c.1126 G > C,het)	SNV	Yes	AD
54	IVD	p.Ala29Thr (c.85G > A,het)	SNV	Yes	AR
**77**	**PKHD1**	**p.Leu2665Pro(c.7994T > C)**	**SNV**	**Yes**	**AR**
**77**	**PKHD1**	**p.Val836Ala(c.2333G > T)**	**SNV**	**Yes**	**AR**
97	TSC2	p.Gly1204Arg(c.3610G > C, het)	SNV	Yes	AD
**Variant with high frequency among healthy people** ^**2**^					
39	GALC	p.Ile562Thr(c.1685T > C, homo)	SNV	No	x 27%
**47**	**GJB2**	**p.Gly114Glu**	**SNV**	**No**	**x 5%**
**47**	**GJB2**	**p.Val27Ile**	**SNV**	**No**	**x 8%**
**Big deletion**					
**7**	**PRODH**	**1 copy del**	**gene del**	**Yes**	**AR**
**7**	**PRODH**	**p.Leu441Pro**	**SNV**	**Yes**	**AR**
15	BTK	del. Exon6~10 (both copies)	partial del	Yes	2 copies
29	DMD	exon2~exon7 duplication	partial dup	Yes	AD
60	MECP2	MECP2 duplication	gene dup	Yes	AD

^1^Heredity = basis for calling disorders; AD = autosomal dominant inheritance; AR = autosomal recessive inheritance, x = No call. Bold indicates a patient with a disorder resulting from composite heterozygotes, phasing issue not resolved but regarded as *trans*; ^2^Disease call was unsuccessful due to a high frequency of alleles among healthy populations.

The analysis algorithm was designed to discover mutations in genes associated with clinical symptoms. Only 5 of 25 (20%) patients showed causal mutations previously associated with genetic disorders that also had information deposited in public databases (Table 3). The other 20 (80%) patients had rare variants with little or no information available. These mutation types included 8 SNVs in 6 (24%) patients, nonsense mutations in 5 (20%) patients, short indels in 3 (12%) patients, and gene deletion or duplication in 4 (16%) patients. These results were a surprise to the analysis team who expected most of the causal mutations to be in public databases, as reported in other cases^20–22^. In addition, compound heterozygosity was the cause of genetic disorders in 7 of 25 (30%) patients. This information was included in the identification of all disorders in our subsequent analysis of the 103 samples.

### Analysis of Results for 103 Participants

We used the same set of algorithms in a blind retrospective validation of variants for all 103 participants. There were 108 predefined causal mutations and 13 negative controls among the 103 participants: 81 patients, 10 carriers, and 12 healthy people. Of the 121 variants in 65 genes, 13 were negative controls, 10 were carrier mutations, and 98 were associated with genetic diseases (Table 4). Of the 81 patients, 57 (70%) had SNV mutations with a dominant disease inheritance and 13 (16%) had mutations for a recessive type of disease inheritance. For patients with two mutations in a single gene, we did not know their phase but hypothesized *tran*s and interpreted it as a compound heterozgote. We detected 115 of the 121 variants (analytical sensitivity 95%, analytical specificity 100%) by analyzing TNGS. We either failed to detect, or excluded, six causal mutations during the selection step. In two patients, sequence reads for the IKBKG gene were too low to detect variants. We checked the BAM file and the sequence alignment file to confirm the absence of sequence reads for this gene. The expansion of trinucleotide repeats in PARM was not detected in one patient, though sequence depth was over 100×. In three patients, variants were detected by TNGS but excluded during final selection of disease-causing mutations because they were in the intron regions and 5 and 14 nucleotides away from splicing sites. We decided to exclude them because there was insufficient information to properly interpret the genetic effects of these mutations. We concluded that we could reliably detect most variants, SNVs and CNVs using TNGS with their current capturing probes, but could not detect the expansion of three nucleotide repeats nor interpret the importance of variants within introns.

**Table 4:** Summary of variant types in 103 participants.

Type of variant	# of samples	# of variants	Relevant genes	Detection
SNV/Trinucleotide expansion	6	6	CYP21A2^1^, IKBKG^2^ PAH^1^, PARMS^3^, VPS33B^1^	Failure
Single Nucleotide Variation (SNV, AD, AR-homo)	54	57		Success
Compound heterozygote	13½ ^4^	27	ABCA12, ACADM, AGL, PRODH, ARSA, ATP7B, GALC, GBA, HEXA, MCCC1, PAH, PKHD1, VPS33B	Success
Copy Number variation (CNV)	7½^4^	8	PRODH, BTK, DMD, MECP2, PMP22, STS	Success
Carrier (het, inheritance type = AR)	10	10	ATP7B, BTK, GALT, GBA, HBB, IVD, LMNA, MCCC2, PRODH, SLC22A5	Success
Negative control	12	13	ABCC8, ACADS, ALPL, CFTR, CYP21A2, GALC, GALT, GJB2, GJB2, NF1, OTC, PMP22, VHL	Success
Total number	103	121		
Predefined variant		121		
Correct answer		115		
Incorrect answer	22	6		
Analytical sensitivity		95%	115/121	

^1^Variants in introns were detected by TNGS, but excluded from disease calling due to ambiguity of their biological roles. ^2^Read depth was zero for IKBKG. ^3^Repeat expansion from 20 to 25 was not detected. ^4^Patient number seven was a heterozygote with two types of variants.

When all 307 genes were considered, each patient had an average of 8.6 (s.d. 0.9) variants that were functionally deleterious according to PolyPhen analysis. These variants occurred at low frequencies (<4%) among healthy people in the 1000 Genome Project (phase 3)^23, 24^ and 4 other Korean populations (JJ, unpublished data). We selected 16 variant positions in 4 patients and resequenced them using the Sanger method. Twelve positions were successfully amplified by PCR and produced sequence information confirming the TNGS results (Supplementary Fig. S1). Based on the selected variants, we predicted all possible disorders in each patient. Literal interpretation of the 8.6 variants resulted in an average of 3.8 putative diseases per patient (Table 2). When we considered a subset of the 65 genes (Supplementary Table S2) responsible for the 57 diseases in 81 patients included in this study, there were on average 1.8 possible genetic disorders in each patient. For the 65 selected genes, 96% of the targeted bases were covered at a minimum depth of 20× reads (Fig. 2). When we reduced the number of genes to those associated with a specific disease, only one or two variants were associated with the selected disease for each patient.

Some patients had known mutations that caused the same amino acid changes as in established pathogenic variants. These mutations were also prevalent in patients affected with dominantly inherited diseases. For these patients, diagnostic evidence was strong for calling a specific genetic disorder, as described in the ACMG guidelines for the interpretation of sequence variants^25^. For example, a patient with the dominantly inherited SNV Gly382Arg (c.1138 G > A, rs28932614) in FGFR3 had the genetic disorder, hypochondroplasia (OMIM 146000). Among 81 patients, only 10 (12%) had mutations known to be associated with genetic disorders (Table 4). Two patients had a recessively inherited causal mutation plus an additional mutation in the same gene. Information on both variants was used to describe the disease as a compound heterozygote. The other 71 (88%) patients had rare mutations and no information was available in OMIM (https://www.ncbi.nlm.nih.gov/omim), ClinVar (https://www.ncbi.nlm.nih.gov/clinvar), or PubMed (https://www.ncbi.nlm.nih.gov/pubmed/) databases. Four of 71 patients each had both copies of a gene deleted, simplifying an association between genes and responsible diseases with strong evidence based on the guidelines^25^. Deletion of both copies through exons 6 to 10 of the ALDH4A1 gene in patient 15 likely caused the genetic disorder, hyperprolinemia (OMIM 942510). Disease association was also relatively straightforward when the variants introduced a premature stop codon either by SNVs or short indels. This occurred even though their association with genetic diseases was not established in public databases. Of the 81 patients, 12 had nonsense mutations by SNVs and 20 had indel mutations. Missense mutations occurred in 34 patients and their impact required cautious interpretation. Another group of 14 patients had mutations at two locations in one gene. For example, one patient was a suspected compound heterozygote for *PAH* (p.Arg243Gln (c.728 G > A rs62508588), (c.442–1 G > A, rs62514907), OMIM 261600), indicative of phenylketonuria disease. Although both mutations were associated with genetic disorders, solving the phase issue of the mutations was technically required to confirm that both copies of the gene were affected. Our decisions were made in this study based on the hypothesis that the mutations were associated with certain diseases. We confirmed our decisions on 14 diseases based on compound heterozygosity when the patients’ information was available. In summary, we were able to identify the causal mutations and correctly diagnose 75 of 81 (92.5%) patients when disease information was available.

## Discussion

The panel used in this study was designed to screen mutations in 307 genes associated with 159 genetic diseases. The list of diseases included 140 rare genetic disorders recommended for prenatal screening by the Health Ministry, ROK, and ~60 neonatal metabolic diseases commonly screened in many hospitals in the ROK. We sequenced 96% of the 3.7-Mb target regions with over 20× coverage in this study and reliably found SNVs, short indels, and CNVs. The sensitivity for finding 121 predefined positions was 95%. Limitations were in failed sequencing, detecting the expansion of trinucleotide repeats, and interpretation of variations within intronic regions (Table 4). We conducted numerous experiments to validate NGS results and Sanger sequencing consistently supported the accuracy of base calls for SNVs and small indels at exon regions (Supplementary Fig. S1; JJ, in preparation). Sequence coverage patterns were consistent among all 103 samples with only 4% of the targeted regions covered by less than 20×. We focused our analyses on the 96% of well-sequence regions. This approach allowed us to multiplex 103 samples and run them in two lanes of HiSeq. 2000.

Among all variants suspected as causal mutations for diverse genetic diseases, only 12% were in public records. There were no or insufficient public records for the other 88%. Our results suggested that screening of previously known causal mutations alone was insufficient and novel variants had to be considered to comprehensively identify causal mutations and reduce false negative results. If the analysis team had ignored the variants not present in public records, the team would have missed diagnosing over 80% of the diseases. We also had to consider that genetic diseases might result from mutations outside of the exome^26^. There are sporadic reports on the roles of introns in regulatory functions and in proper gene splicing^27^. Informal polling among geneticists suggested that exome-based discovery of causative mutations identifies less than 50% of cases^28^. Thus it may be necessary to sequence whole genic regions, including complete introns and regulatory regions. The cost of TNGS is now low enough that adding introns would not substantially increase the price. It would still be challenging, however, to understand the effect of mutations in introns; we failed to predict their deleterious effects in three cases even after detecting their presence.

We primarily evaluated the accuracy of TNGS in detecting variants and in discovering disease-causing variants among them without prior information on causal mutations. We predicted the candidates of causal variants for 307 genes without knowing the name of the disease. We then made predictions knowing the disease names associated with each sample. When the disease names were not known, we attained multiple causative variants for several putative diseases with similar weight in each patient. A large amount of sequence information was available for several populations in the world^23, 24, 29, 30^. Although the information on allele frequencies of genetic variants was insufficient to distinguish extremely rare causal mutations from low-frequency polymorphisms, it was a very effective tool to reduce the number of candidate variants (see below).

PolyPhen alone predicted more than one disease, including one that was patient specific. On average 75 variants were predicted by PolyPhen as having damaging effects. The sensitivity was close to 95%, but specificity was low due to the over-prediction of missense variants as pathogenic mutations. Our results were consistent with knowledge that algorithms either predict missense variants or over-predict as known disease variants with 65–80% accuracy^31, 32^. We could have improved the program after the initial tests, but it seemed apparent that the pathogenic variants detected by PolyPhen were only candidates of causal variants and not truly pathogenic variants. It is of note that the list of candidate variants was reduced from 75 to 8.6 on average when we removed variants with allele frequencies over 4% among healthy populations. The list of candidate variants associated with the disease in each patient became apparent when we incorporated the disease names into the analysis. The penetration rate of causal variants can vary depending on genetic background, regardless of their extremely low frequency^33^. Thus, additional information is needed to unequivocally establish a cause-and-effect relationship between a mutation and a disease, This information could include: 1) sequence data for the unaffected parents and the affected child, 2) the same mutations in an affected sibling and the unmutated genes of a healthy sibling, and 3) at least two cases of independent mutations in one gene^34^. We have been working on several cases to establish a cause-and-effect relationship between mutations and diseases (JSL, in preparation).

Next-generation sequencing is powerful enough to be used for primary screening of rare genetic disorders. There have been several attempts to use NGS as the initial screening method in the Republic of Korea. Health Insurance Review and Assessment Services in the ROK decided to cover the diagnostic services and reimburse the associated expense from March 1, 2017. However, three major issues must be addressed to make these services sustainable: reasonable turnaround time, affordable cost, and proper interpretation of variants^17^.

Turnaround time and cost are two intertwined issues. NGS delivers large amounts of sequence data, but has been too expensive to routinely apply to neonatal screening of genetic disorders. The read number and cost of sequencing runs are fixed regardless of the sample numbers. The technology would be cost effective if TNGS was combined with the parallel processing of sequencing reactions. For example, if 48 samples were run together, TNGS of a 1-Mb region with a 1-Gb output would probably cover ~500× reads with a total sequencing cost of less than $400. The demand for testing individual genetic diseases is likely too low to take advantage of the parallel processing of multiple samples. Individual diseases are rare, usually less than 1 in 10,000^35^. The expected incidence of Wilson’s disease, for example, was 1 in 36,000 newborns, or about 10 patients each year in the ROK^36^. Over 5,000 genetic diseases caused by SNVs, short indels, or CNVs are recorded in the OMIM database (https://www.ncbi.nlm.nih.gov/omim)^37^. Test panels examining numerous genes and CNVs affecting over 100 diseases instead of just a few, would be clinically valuable and economically viable. Parallel computation and interpretation of data appeared to solve another cost issue for many patients that arose from the analysis of NGS data. It took about 5 hours instead of 58 days to identify rare variants in 103 samples when 200 nodes of a supercomputer were used for the analysis (Table 1). A company with the computation capacity could serve as a Biological Computation Center to hospitals with small-scale computation facilities. Computation time would not be a bottleneck for NGS analysis used in disease screening even if numerous sequences were multiplexed. Any panel design to detect both SNVs and CNVs would further expand the coverage of genetic diseases. This could increase demand for the panel, shorten turnaround time, and lower cost.

When many genes are examined in a single test, more rare variants can be screened and considered for their possible association to disease. When screening variants in patients with accurate phenotypic characterizations, few candidates among the over 5,000 diseases in the OMIM database would help identify the narrow set of candidate genes for a disease. An expanded range of candidate genes might increase the odds of identifying the causal variants. If TNGS were used to screen for neonatal diseases before symptoms appeared, all variants in all genes could be considered as candidates of disease-causing mutations. If rare variants with insufficient public records were excluded from the screening, over 80% of potential diseases would not be reported, producing false negative results. It would be ethically and scientifically inappropriate not to report all rare variants. Conversely, reporting all variants would lead primary doctors and parents into a dilemma, if not panic. They would be faced with an avalanche of data without knowing the possible implications. In this study, 8.6 variants were associated with 3.4 diseases in each person when 307 genes were analyzed. About 88% of the variants were uncommon mutations among healthy people and not causing genetic diseases. Therefore, appropriate notice or instruction must be given so physicians can review family histories and check patients for symptoms of known genetic diseases. TNGS could provide nationwide screening for genetic diseases caused by SNVs and CNVs in the near future. A collective scientific effort is needed to develop TNGS as a primary screen for rare genetic diseases.

## Materials and Methods

### Target Genes for NGS

We developed clinical test panels to screen for rare mutations associated with 159 genetic diseases (Supplementary Table S1). A targeted gene enrichment method was used to construct libraries for subsequent determination of sequences using a next-generation sequencing (NGS) method with HiSeq (Illumina, San Diego, California). The probe set was designed to capture 3.7 megabases (Mb) covering the exonic regions and 25 nucleotides at the flanking intronic areas for 362 targeted genes plus 13,000 biologically important locations. Probe-library hybridization followed by capturing target genes was performed according to the manufacturer’s instructions. A list of the 362 genes examined in this study is shown in Supplementary Table S1.

### Sample Preparation

We used DNA samples from 103 participants, including 81 patients with clinical symptoms previously monitored and diagnosed at Yonsei Severance General Hospital, ROK. The study was approved by the institutional review board and the ethics committee of the hospital. All methods were performed in accordance with the relevant guidelines and regulations as described below. Written informed consent for genetic testing, or a waver, was obtained from each participant or family. All samples were randomly numbered and processed in accordance with hospital guidelines, from library construction to subsequent sequencing and analysis. Samples from patients were collected over a 10-year period from 2006 to 2015. DNA was extracted from a 200-µl blood sample with the QIAcube System and QIAamp DNA Blood Mini Extraction Kit (Qiagen, Valencia, California) and stored in 10 mM Tris buffer solution at −20 °C. DNA extraction, library construction and HiSeq. 2000 (Illumina, San Diego, California) running were performed at the Clinical Genetics Laboratory, Yeonsei University (Seoul, ROK).

### Library Preparation and Sequencing

DNA quality was evaluated when it was quantitated with 4200 TapeStation (Agilent, UK). A sequencing library was constructed with 500 ng of acceptable-quality genomic DNA, as described below. We fragmented the DNA into 150- to 200-base-pair (bp) segments with a NEBNext dsDNA Fragmentase® (New England Biolabs, UK), according to the manufacture’s protocol. The enzyme-fragmented DNA was subsequently used to construct a library according to the protocol provided by Cellemix (Seoul, ROK). Briefly, the library was constructed by repairing blunt ends, adenylating the 3′ ends, and ligating the adapters. The adapter-ligated library was amplified using six cycles of polymerase chain reaction. The concentration and clone-size of each library were examined at this stage. For the enrichment of the library, we used a custom probe set designed by Syntekabio, Inc. (Daejeon, ROK) and synthesized by Cellemix (Seoul, ROK).

Following the capturing procedure, the samples were tagged with index primers using 24 cycles of post-capture amplification. The concentration and clone size of each amplified library were then examined. A total of 96 samples with different index tags were pooled and sequencing performed on a HiSeq. 2000. Each sample generated 150-bp, paired-end sequencing reads. To minimize lane-to-lane variation, an equal amount of indexed library was combined before the sequence run.

### Analysis of Sequence and Detection of Variants

We analyzed the NGS data using a series of algorithms in a predetermined order (Fig. 1). Raw image files were converted to base calls by real-time analysis on HiSeq, using the default settings recommended by the manufacturer (Illumina, San Diego, CA). The output base call files (*.bcl) generated by the real-time analysis were converted to FASTQ files with consensus assessment of sequence and variation (CASAVA pipeline, version 1.8, Illumina). CASAVA also demultiplexed the data to obtain FASTQ files for individual samples. The FASTQ files were sent to Syntekabio, Inc. for sequence alignment and further analysis (Fig. 1). A Burrows-Wheeler aligner (BWA-MEM; version 0.7.10^38^) was used with default parameters to align the sequence reads to the human reference genome sequence GRCh37. Alignments in the sequence alignment/map (SAM) format were converted to binary alignment map (BAM) files implemented in SAM tools (SAM tools version 0.1.10^39^). Picard tool (version 1.119; http://picard.sourceforge.net) was used to remove duplicate reads and to sort sequence reads in the order of their start position. BAM files were realigned to the reference sequences Ch37d.5.fa with GATK Realigner Target Creator and local alignment was fine-tuned with GATK Indel Realigner. The GATK base-quality recalibration tool, GATK Base Recalibrator, was used to recalibrate base quality scores. Subsequently, a fine-tuned BAM file generated by GATK PrintReads was used for variant calling. For the above described analyses, we used algorithms imbedded in GATK software tools (version 3.3.0; http://www.broadinstitute.org)^40^. CNVnator^41^ and Contra^42^ were used to detect copy number variations.

### Coverage Analysis

Before discovering single nucleotide variants (SNVs), indels, and copy number variants (CNVs), we analyzed coverage in the targeted exons and estimated target enrichment. To compute minimum, maximum, and average coverage of each exon, BAM files containing coverage data were first converted to pileup with SAM tools. We recalibrated the BAM file as described above. Subsequently, Picard CalculateHsMetrics tools (Broad Institute, https://broadinstitute.github.io/picard/) was used to calculate on-target rates. The program generated a metrics file containing “on-target bases” and “mean coverage (depth)” on the bed file. On-target rates were calculated by the equation, on-target bases/total bases.

### Analysis of Variants

The analysis team investigated whether the variations in question were previously reported by searching local databases reflecting the Online Mendelian Inheritance in Man (OMIM, NCBI, http://omom.org, 09–01–2016) and ClinVar (https://www.ncbi.nlm.nih.gov/clinvar/)^43^. For previously published variants, information on OMIM, ClinVar, and PubMed (US National Library of Medicine, Bethesda, Maryland) was consulted to determine the functional significance of the identified variants and select the candidates of causative variations for diseases. We evaluated the functional importance of novel variants using the software PolyPhen (ver. 2.2.2)^44, 45^. For each variant, regardless of its functional importance, we calculated its frequency at each position among the population to evaluate the prevalence of the identified variants in human populations. We used the following databases: the 1000 Genomes Project (phase 3)^23, 24^, the dbSNP database (build version 144, NCBI, Bethesda, Maryland), 2000 people in the International Cancer Genome Consortium^29, 30^, the 1000 Korean Genome, and the 500 healthy babies (JJ, unpublished data) database. Large deletions that may have resulted from hemizygous or composite heterozygous mutations were not distinguished in this study. We built and used local databases for the OMIM, ClinVar, and PolyPhen results, and allele frequencies in different populations when classifying the functional importance of each variant.

### Verification of Mutations by Sanger Sequencing

All positions associated with genetic diseases were sequence-verified by the Sanger method. In addition to positions showing causal mutations, we also determined the sequences of other positions in four samples with rare genetic variations. For Sanger sequence verification, we performed PCR amplification using 10ng of genomic DNA. Primers for the PCR reactions were designed using NCBI/Primer-BLAST (http://www.ncbi.nlm.nih.gov/tools/primer-blast/). The size of amplified PCR products was designed to be in the range of 414 to 681 bp (Supplementary Table S4). For PCR amplification of the fragments, H-Star Taq DNA Polymerase (BIOFACT Co. Ltd, Daejeon, Korea) was used. PCR products were purified with a Gel & PCR Purification Kit (BIOFACT Co. Ltd, Daejeon, Korea) and used as a template for Sanger sequencing. Chromatograms were manually inspected to confirm the sequence accuracy of each file. The information on chromosome positions of variants, sequence of each primer, and amplicon sizes is provided in Supplementary Table S4.

### Analytical Validation

Analytical sensitivity and specificity were respectively calculated by the formulas, TP/(TP + FN) and TN/(TN + FP), where TP, TN, FP, and FN were respectively true positive, true negative, false positive, and false negative. Sequence variants identified through the analysis of NGS data were compared to the predefined variants determined by Sanger sequencing. We included 121 positions in the calculation of true positive, true negative, false positive, and false negative. True positives were identically sequenced as positive at the 121 positions by both the Sanger and NGS methods. True negatives were the 120 positions for each patient and included all 121 sequenced positions except for a causative variant; they were negative by both the Sanger and NGS methods. False positives were any of the 121 positions with the wrong sequence and were negative by the Sanger method and positive by NGS. False negatives were wrong sequences for the 121 positions and were positive by the Sanger method and negative by NGS.

### Data Availability

All data analysed during this study are included in this published article and its Supplementary Information files. The raw NGS datasets generated during the current study are not publicly available due to restrictive hospital policies but are available from the corresponding author on reasonable request.

## Electronic supplementary material

Supplementary Table 1

Supplementary Table 2

Supplementary Table 3

Supplementary Table 4
